# Alkaloids and Phenolic Compounds from *Sida rhombifolia* L. (Malvaceae) and Vasorelaxant Activity of Two Indoquinoline Alkaloids

**DOI:** 10.3390/molecules22010094

**Published:** 2017-01-06

**Authors:** Otemberg Souza Chaves, Yanna Carolina Ferreira Teles, Matheus Morais de Oliveira Monteiro, Leônidas das Graças Mendes Junior, Maria de Fátima Agra, Valdir de Andrade Braga, Tânia Maria Sarmento Silva, Maria de Fátima Vanderlei de Souza

**Affiliations:** 1 Post Graduation Program in Bioactive Natural and Synthetic Products, Health Sciences Center, Federal University of Paraíba, 58051-970 João Pessoa, PB, Brazil; otembergsc@ltf.ufpb.br (O.S.C.); monteirommo@gmail.com (M.M.d.O.M.); leonidasjunior@ltf.ufpb.br (L.d.G.M.J.); agramf@ltf.ufpb.br (M.d.F.A.); valdir@cbiotec.ufpb.br (V.d.A.B.); 2 Post Graduation in Development and Technological Innovation in Medicines, Federal University of Paraiba, 58051-900 João Pessoa, PB, Brazil; yannateles@gmail.com; 3 Department of Chemistry and Physics, Agrarian Sciences Center, Federal University of Paraíba, 58397-972 Areia, PB, Brazil; 4 Department of Molecular Sciences, Federal Rural University of Pernambuco, Campus Dois Irmãos, 52171-900 Recife, PE, Brazil; sarmentosilva@gmail.com

**Keywords:** *Sida rhombifolia*, Malvaceae, indoquinoline alkaloids, vasorelaxant activity

## Abstract

The follow-up of phytochemical and pharmacological studies of *Sida rhombifolia* L. (Malvaceae) aims to strengthen the chemosystematics and pharmacology of *Sida* genera and support the ethnopharmacological use of this species as hypotensive herb. The present work reports phytoconstituents isolated and identified from aerial parts of *S. rhombifolia* by using chromatographic and spectroscopic methods. The study led to the isolation of scopoletin (**1**), scoporone (**2**), ethoxy-ferulate (**3**), kaempferol (**4**), kaempferol-3-*O*-β-d-glycosyl-6′′-α-d-rhamnose (**5**), quindolinone (**6**), 11-methoxy-quindoline (**7**), quindoline (**8**), and the cryptolepine salt (**9**). The alkaloids quindolinone (**6**) and cryptolepine salt (**9**) showed vasorelaxant activity in rodent isolated mesenteric arteries.

## 1. Introduction

The Malvaceae family Juss. has predominantly pantropical distribution, including about 250 genera and 4200 species [1]. In Brazil, 70 genera are found widely spread, from which nine are considered endemic [2]. The *Sida* genus is considered native to Brazil and presents 95 species [3]. Many *Sida* species are used in American, African, and Asian countries, showing good efficacy in health disorders [4].

*Sida rhombifolia* L. is popularly known in Brazil as “matapasto”, “guanxuma”, and “relógio”. It is used in Indian popular medicine against hypertension, diabetes, and for the treatment of gout [5,6]. Previous studies have reported chemical constituents isolated from this species, including steroids, chlorophyll derivatives, flavonoids, alkaloids, β-phenylethylamines, and carboxylated tryptamines [4,5].

In order to strengthen chemosystematics and pharmacology of *Sida* genera, as well as to support the popular medicinal use of this plant, the phytochemical study of *S. rhombifolia* (Malvaceae) was continued. The study led to the isolation of nine secondary metabolites from the aerial parts of *S. rhombifolia.* Additionally, the vasorelaxant activity of two isolated alkaloids was demonstrated, justifying the popular use of the species against arterial hypertension.

## 2. Results

### 2.1. Identification of Isolated Compounds

**Figure 1:**
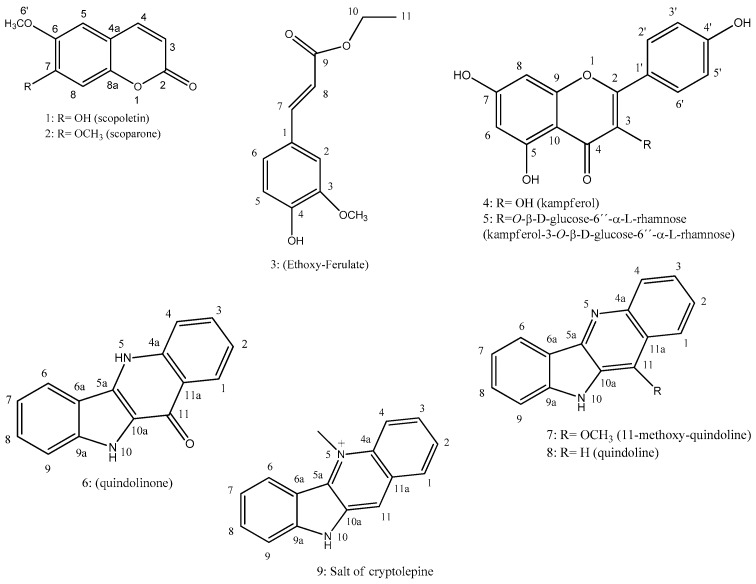
Structure of compounds isolated from *S. rhombifolia* (Malvaceae).

The isolated and identified compounds from aerial parts of *S. rhombifolia* are showed in Figure 1. The NMR data of compound **7** (^1^H, ^13^C, Heteronuclear Single Quantum Correlation-HSQC, Heteronuclear Multiple Bond Correlation-HMBC, and Nuclear Overhauser Spectroscopy-NOESY) are presented in Table 1. Spectral data from the other compounds are also presented.

#### Spectral Data

2*H*-*1-Benzopyran-2-one, 7-hydroxy-6-methoxy* (Scopoletin, **1**). Colourless crystal: ^1^H-NMR (500 MHz) (CD_3_OD) δ: 6.21 (d, *J* = 9.4 Hz, 1H, H-3), 7.86 (d, *J* = 9.4 Hz, 1H, H-4), 7.12 (s, 1H, H-5), 6.77 (s, 1H, H-8) and 3.91 (s, 3H, H-6′). ^13^C-NMR (125 MHz) (CD_3_OD) δ: 164.0 (C-2), 112.6 (C-3), 146.0 (C-4), 112.5 (4a), 110.0 (C-5), 147.0 (C-6), 152.9 (C-7), 103.9 (C-8), 151.4 (8a) and 56.8 (6′-OCH_3_). In agreement with literature [7].

*2H*-*1-Benzopyran-2-one, 6,7-dimethoxy* (Scoparone, **2**). Colourless crystal: ^1^H-NMR (200 MHz) (CDCl_3_) δ: 6.25 (d, *J* = 9.48 Hz, 1H, H-3), 7.37 (d, *J* = 9.48 Hz, 1H, H-4), 6.83 (s, 1H, H-5), 6.81 (s, 1H, H-8), 3.92 (s, 3H, 6′-OCH_3_) and 3.89 (s, 3H, 7′-OCH_3_). ^13^C-NMR (50 MHz) (CDCl_3_) δ: 161.4 (C-2), 113.4 (C-3), 143.3 (C-4), 111.3 (4a), 107.8 (C-5), 146.2 (C-6), 152.7 (C-7), 99.9 (C-8), 149.9 (C8a), 56.36 (6′-OCH_3_) and 56.32 (7′-OCH_3_). In agreement with literature [8].

*2-Propenoic acid, 3-(4-hydroxy-3-methoxyphenyl)-ethyl ester* (Ethoxy-ferulate, **3**). Yellowish oil: ^1^H-NMR (500 MHz) (CDCl_3_) δ: 7.03 (d, *J* = 2.0 Hz, 1H, H-2), 6.91 (d, *J* = 8.15Hz, 1H, H-5), 7.07 (dd, *J* = 8.15; 2.0 Hz, 1H, H-6), 7.61 (d, *J* = 15.9 Hz, 1H, H-7), 6.29 (d, *J* = 15.9 Hz, 1H, H-8), 4.26 (q, *J* = 7.15 Hz, 2H, H-10), 1.33 (t, *J* = 7.15 Hz, 3H, H-11) and 3.92 (s, 3H, 3′-OCH_3_). ^13^C-NMR (125 MHz) (CDCl_3_) δ: 127.2 (C-1), 109.4 (C-2), 146.9 (C-3), 148.0 (C-4), 114.8 (C-5), 123.1 (C-6), 144.7 (C-7), 115.8 (C-8), 167.4 (C-9), 60.5 (C-10), 14.5 (C-11) and 56.10 (3′-OCH_3_). In agreement with literature [7].

*4H-1-Benzopyran-4-one, 3,5,7-trihydroxy-2-(4-hydroxyphenyl)* (Kaempferol, **4**). Yellow powder: ^1^H-NMR (500 MHz) (CD_3_OD) δ: 6.18 (d, *J* = 2.00 Hz, 1H, H-6), 6.39 (d, *J* = 2.00 Hz, 1H, H-8), 8.08 (d, *J* = 8.9 Hz, 2H, 2′/6′) and 6.90(d, *J* = 8.9 Hz, 2H, 3′/5′). ^13^C-NMR (125 MHz) (CD_3_OD) δ: 148.0 (C-2), 137.1 (C-3), 17.3 (C-4), 162.5 (C-5), 99.3 (C-6), 165.6 (C-7), 94.4 (C-8), 158.2 (C-9), 103.7 (C-10), 123.7 (C-1′), 130.6 (C-2′/C-6′), 116.3 (C-3′/C-5′) and 160.5 (C-4′). In agreement with literature [9].

*4H-1-Benzopyran-4-one, 3-[[6-*O*-(6-deoxy-α-l-mannopyranosyl)-β-d-galactopyranosyl]oxy]-5,7-dihydroxy-2-(4-hydroxyphenyl)* (Kaempferol-3-*O*-β-d-glucose-6′′-α-d-rhamnose, **5**). Yellow powder: ^1^H-NMR (500 MHz) (CD_3_OD) δ: 6.16 (d, *J* = 2.0 Hz, 1H, H-6), 6.34 (d, *J* = 2.0 Hz, 1H, H-8), 8.04 (d, *J* = 8.89 Hz, 2H, 2′/6′), 6.87 (d, *J* = 8.89 Hz, 2H, 3′/5′), 5.08 (d, *J* = 7.48 Hz, 1H, H-1′′), 3.38-3.82 (m, 6H, H-2′′ a H-6′′), 4.50 (bs, 1H, H-1′′′), 3.38-3.82 (m, 5H, H-2′′′ a H-5′′′) and 1.12 (d, *J* = 6.08 Hz, 3H, H-6′′′). ^13^C-NMR (125 MHz) (CD_3_OD) δ: 159.4 (C-2), 137.4 (C-3), 179.3 (C-4), 162.8 (C-5), 100.0 (C-6), 166.6 (C-7), 94.9 (C-8), 158.5 (C-9), 105.5 (C-10), 122.6 (C-1′), 132.3 (C-2′/6′), 116.1 (C-3′/5′), 161.4 (C-6′), 104.5 (C-1′′), 71.3 (C-2′′), 78.0 (C-3′′), 75.6 (C-4′′), 69.7 (C-5′′), 68.5 (C-6′′), 102.3 (C-1′′′), 72.0 (C-2′′′), 72.2 (C-3′′′), 77.1 (C-4′′′), 73.8 (C-5′′′) and 17.9 (C-6′′′). In agreement with literature [10].

*11H-Quindolin-11-one-5,10-dihydro* (Quindolinone, **6**). Yellow powder: ^1^H-NMR (500 MHz) (DMSO-*d*_6_) δ: 8.35 (dd, *J* = 8.1, 1.2 Hz, 1H, H-1), 7.28 (td, *J* = 8.1; 6.8; 1.0 H z, 1H, H-2), 7.68 (td, *J* = 8.3; 6.8; 1.57 Hz, 1H, H-3), 7.73 (dd, *J* = 8.3; 1.0 Hz, 1H, H-4), 12.43 (s, 1H, 5-NH), 8.19 (dl, *J* = 8.0 Hz, 1H, H-6), 7.20 (td, *J* = 8.0; 6.8; 1.0 Hz, 1H, H-7), 7.47 (td, *J* = 8.3; 6.8; 1.2 Hz, 1H, H-8), 7.52 (dt, *J* = 8.3; 1.0 Hz, 1H, H-9) and 11.67 (s, 1H, 10-NH). ^13^C-NMR (125 MHz) (DMSO-*d*_6_) δ: 125.1 (C-1), 120.4 (C-2), 130.6 (C-3), 117.7 (C-4), 139.0 (C-4a), 128.8 (C-5a), 115.8 (C-6a), 120.7 (C-6), 118.8 (C-7), 127.3 (C-8), 112.5 (C-9), 138.5 (C-9a), 123.0 (C-10a), 167.3 (C-11) and 122.8 (C-11a). In agreement with literature [11].

*10H-Quindoline, 11-methoxy* (11-Methoxy-quindoline, **7**). Yellow powder: Table 1. In agreement with literature [12]. 

*10H*-Quindoline (Quindoline, **8**). Orange crystal. High Resolution Mass Spectrometry by Electronspray (HRESI) (+)-MS *m*/*z* 219.09 (calc. for C_15_H_10_N_2_: 218.08 g/mol). ^1^H-NMR (500 MHz) (DMSO-*d*_6_) δ: 7.63 (d, *J* = 10.0 Hz, 1H, H-1), 7.39 (td, *J* = 10.0 Hz, 1H, H-2), 7.67 (td, *J* = 10.0 e 5.0 Hz 1H, H-3), 8.29 (d, *J* = 10.0 Hz, 1H, H-4), 8.44 (d, *J* = 10.0 Hz, 1H, H-6), 7.24 (td, *J* = 10.0; 5.0Hz, 1H, H-7), 7.57 (m, 2H, H-8 e H-9), 11.72 (s, 1H, 10-NH) and 8.52 (s, 1H, H-11). In agreement with literature [12].

*10H-Quindolinium, 5-methyl* (Salt of cryptolepine, **9**): NMR data published [5].

### 2.2. Pharmacological Data

**Figure 2:**
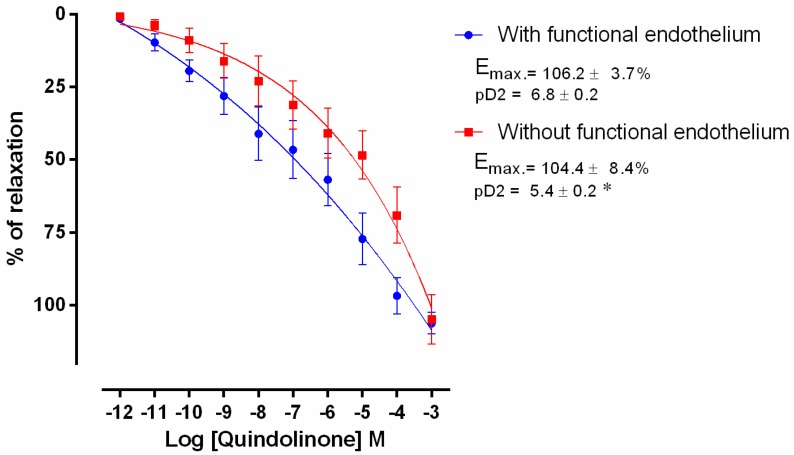
Concentration-response curve of quindolinone (10^−12^–10^−3^ M) in cranial mesenteric artery rings isolated from rats with functional endothelium (•) and without functional endothelium (■) pre-contracted with phenylephrine (PHE, 1 μM). Values are expressed as mean ± standard error of mean (SEM), * *p* < 0.05 compared to the ring with functional endothelium.

**Figure 3:**
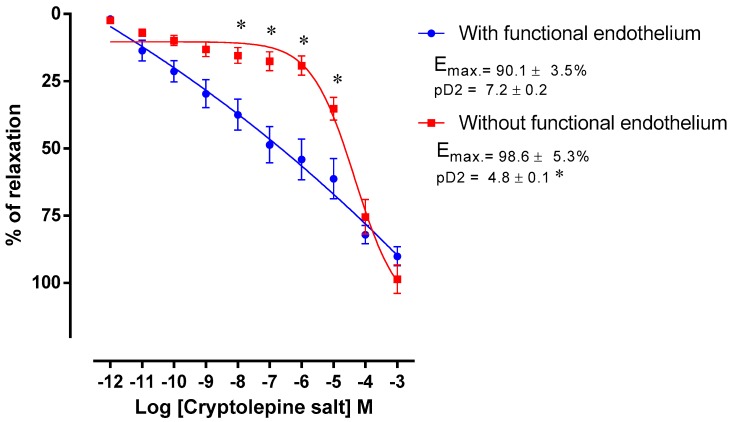
Concentration-response curve of cryptolepine salt (10^−12^–10^−3^ M) in cranial mesenteric artery rings isolated from rats with functional endothelium (•) and without functional endothelium (■) pre-contracted with phenylephrine (PHE, 1 μM). Values are expressed as mean ± standard error of mean (SEM), * *p* < 0.05 compared to the ring with functional endothelium.

The vasorelaxant activity of compounds **6** (quindolinone) and **9** (salt of cryptolepine) were evaluated by increasing cumulative addition of the compound (10^−12^–10^−3^ M) using rodent mesenteric arteries pre-contracted with phenylephrine (PHE) (1 μM). The experiments were carried out using arteries with functional endothelium and without functional endothelium (Figure 2 and Figure 3). Data were expressed as maximum effect (Emax) and the potency of the drug was expressed as pD_2_, which is the log of the 50% of the effective concentration (EC_50_).

## 3. Discussion

The ^1^H-NMR spectra of compound **7** showed signals with a pattern of substitution indicating an indoquinoline skeleton [5], aromatic rings ortho di-substituted with the presence of four broad doublets : δ_H_ 8.37 *J* = 7.85 Hz (H-6); δ_H_ 8.30 *J* = 8.45 Hz (H-1); δ_H_ 8.18 *J* = 8.5 Hz (H-4); δ_H_ 7.64 *J* = 7.1 Hz (H-9), besides four broad triplets: δ_H_ 7.70 *J* = 7.45 Hz (H-3); δ_H_ 7.63 *J* = 7.6 Hz (H-8); δ_H_ 7.58 *J* = 7.55 Hz (H-2); δ_H_ 7.30 *J* = 7.0 Hz (H-7) (Table 1). 

A singlet at δ_H_ 11.50 (1H) was detected indicating the N-H of indol (NH-10) found in this group of alkaloids [5]. The hydrogens at δ_H_ 4.39 (s, 3H) were attributed to a methoxy group bonded to C-11, since the chemical shift at δ_H_ 9.02 characteristic of H-11 was not found [12]. 

The ^13^C-NMR data supported the proposal of indoquinoline nucleous for compound **7** by showing signals for 15 carbons. The chemical shift at δ_C_ 60.7 indicated a methoxy group as proposed by ^1^H-NMR, being confirmed by the HMQC (Table 1).

**Figure 4:**
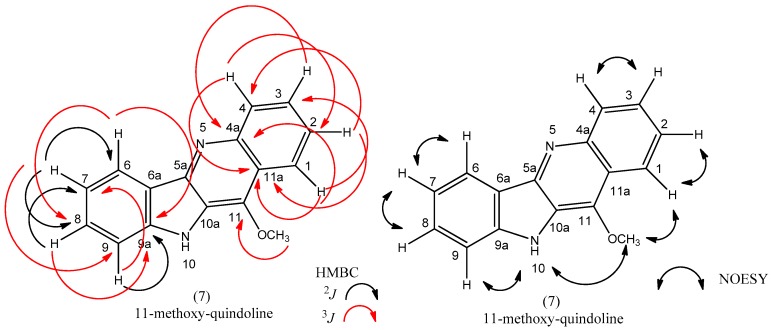
HMBC and NOESY correlations for compound **7**.

The HMBC spectrum of **7** allowed establishing the chemical shifts of C-4a, C-6a, C-9a, C-11, and C-11a. Relevant correlations could be seen between: H-3 and H-1 with C-4a (*J*^3^); H-9 with C-6a (*J*^3^); H-8 with C-9a (*J*^3^) and H-1 with C-9a (*J*^2^) (Table 1 and Figure 4).

The NOESY spectra of compound **7** showed correlations between neighbour hydrogens, as well as confirmed that the methoxy is bonded to C-11, by showing correlation between δ_H_ 4.39(O-CH_3_) with δ_H_ 11.50 (NH-10) and δ_H_ 8.30 (d, H-1).

The tested substances quindolinone (**6**) and the salt of cryptolepine (**9**) promoted vasorelaxation in mesenteric artery rings with functional endothelium and after endothelium removal. The PHE potency in promoting arterial contraction was significantly reduced by compounds **6** and **9** (quindolinone: 6.8 ± 0.2 vs. 5.4 ± 0.2, *p* <0.05 and salt of cryptolepine: 19.7 ± 0.2 vs. 4.77 ± 0.1, *p* < 0.05) (Figure 2 and Figure 3). 

PHE is an agonist of α1- adrenergic receptors [13,14], whose are coupled to Gq/11 protein. Once activated, the receptors trigger the hydrolysis of phosphatidyl inositol 4,5-bisphosphate (PIP_2_) into inositol 1,4,5-trisphosphate (IP_3_) and diacylglycerol (DAG) by the action of phospholipase C-β (PLCβ). Then, the IP3 receptors at the sarcoplasmic reticulum are activated, releasing Ca^2+^ into the cytosol. DAG and the elevation of intracellular Ca^2+^ activate kinase proteins, including protein kinase C (PKC) in smooth muscle. It results in phosphorylation and inactivation of enzymes related to the contractile process, such as myosin light chain phosphatase (MLCP) [15,16]. 

After endothelium removal, the vasorelaxant effects of both tested compounds were significantly altered. The vascular endothelium secretes several biologically active substances, and many of them are active in vessels’ muscle cells, modulating the vascular tone. The vasodilator mechanism of endothelium is mediated by endothelium*-*derived relaxing factor (EDRF), such as nitric oxide (NO), prostacyclin, bradykinin, endothelium-derived hyperpolarizing factor (EDHF), and metabolites of monooxygenases, among others [17,18,19,20]. On the other hand, the endothelium*-*derived constrictor factors (EDCF) promote contraction, i.e., angiotensin II, endothelin, vasoconstrictors prostanoids, and reactive oxygen species, such as superoxide anion [21,22].

Thus, the vasorelaxant activity of tested compounds seems to involve endothelium-derived substances, since the effect was decreased after the endothelium removal. However, the compounds also seem to act in smooth muscle cells, since in endothelium-free preparations it was possible to observe some vasorelaxant effect. The vasorelaxant effect of cryptolepinone isolated from *S. rhombifolia* has been previously reported [5].

## 4. Materials and Methods

### 4.1. General 

Silica gel 60 (Merck, Rio de Janeiro, RJ, Brazil) 7734 (0.063–0.2 mm particle, 70–230 mesh), neutral alumina, Sephadex LH-20, and XAD-2 were used for the chromatographic procedures. ^1^H- and ^13^C-NMR spectra were recorded on a Varian Oxford 200 NMR spectrometer (200/50 MHz, Varian, São Paulo, Brazil) and on a Varian 500 NMR spectrometer (500/125 MHz, Varian)*.* Mass spectra were obtained on a Xevo*^®^* G2*-*XS QT of mass spectrometer (Waters, Campinas, Brazil). 

### 4.2. Collection, Extraction, and Isolation

*Sida rhombifolia* was collected in Santa Rita city (PB, Brazil) in September 2008 and identified by Dr. Maria de Fátima Agra. A voucher specimen (Agra 7045) is kept at the herbarium Professor Lauro Pires Xavier (CCEN/UFPB). The dried and powdered aerial parts of the plant (5.5 kg) were extracted with 95% EtOH (10 L, room temperature) for 72 h. The EtOH extract was concentrated under reduced pressure at 40 °C, providing 570 g of crude ethanol extract (CEE).

The CEE of *S. rhombifolia* showed positive results when tested with Dragendorff, Bouchadart, and Mayer reagents for detection of alkaloids. Thus, the classic alkaloid extraction was performed using 100 g of the CEE.

The acid CHCl_3_ fraction (1.2 g) was subjected to silica gel column chromatography (CC) using hexane (Hex), dichloromethane (CH_2_Cl_2_), and methanol (MeOH), alone or in binary mixtures of increasing polarity, resulting in the isolation of compounds **1** (21 mg), **2** (9 mg), and **3** (8 mg). The total alkaloids fraction (800 mg) was submitted to CC using neutral alumina as stationary phase. The column fraction 14/43 (28 mg) was chromatographed on preparative, Analytical Thin Layer Chromatography (TLC) using alumina and eluted with Hex:ethyl acetate (8:2), yielding the pure compounds **7** (12 mg) and 8 (5 mg).

Part of the CEE (200 g) was subjected to filtration under reduced pressure (VLC) using silica gel 60 as stationary phase and eluted with hexane (Hex.), ethyl acetate (EtOAc), and methanol (MeOH) alone or in binary mixtures increasing polarity.

An sample of the MeOH phase (10 g) was submitted to CC on XAD-2 using the eluents: H_2_O, H_2_O:MeOH (7:3), H_2_O:MeOH (1:1), MeOH, Hex, acetone, and ethyl acetate (AcOEt). Fractions of MeOH (162 mg) and H_2_O:MeOH (7:3) (475 mg) were fractioned on a Sephadex LH-20 (MeOH) leading to isolation of compounds **4** (5 mg) and **5** (10 mg).

Another part of the CEE (200 g) was dissolved in EtOH–H_2_O (9:1) and submitted to liquid-liquid chromatography using Hex, CH_2_Cl_2_, EtOAc and *n*-butanol, obtaining the respective phases. The CH_2_Cl_2_ phase (26 g) was chromatographed on VLC (silica gel 60) eluted with CH_2_Cl_2_ and MeOH alone and in mixtures increasing polarity. The fraction CH_2_Cl_2_:MeOH (9:1) (7 g) was chromatographed on silica gel CC using the same solvents. The fraction 66/74 (85 mg) was purified on a Sephadex LH-20 (MeOH) yielding compound **6** (23 mg).

### 4.3. Biological Assay

The experiments were approved by the Federal University of Paraíba Ethical Committee for Animal Use under the protocol CEPA#305/09. After animal euthanasia by decapitation using a guillotine, the cranial mesenteric artery was isolated, placed in Tyrode’s solution and dissected in order to make it free of adhering tissue. In endothelium-denuded experiments, endothelium was removed by rubbing the intimal surface of the vessels. Rings with 1–2 mm were obtained and placed in physiological Tyrode’s solution, maintained to 37 °C, gassed with carbogenic mixture (95% O_2_ and 5% CO_2_), and kept at pH 7.4. All preparations were stabilised under a resting tension of 0.75 g for 1 h. The solution was replaced every 15 min in order to prevent the accumulation of metabolites. The force of contraction was isometrically recorded by a force transducer (Miobath-4, WPI, Sarasota, FL, USA) coupled to an amplifier-recorder (Miobath-4) and to a computer equipped with an analog-to-digital converter board as described earlier. The presence of functional endothelium was assessed by the ability of acetylcholine (10 µM) to induce more than 80% relaxation of vessels pre-contracted with phenylephrine (10 µM). Less than 10% of relaxation to acetylcholine was taken as evidence that the vessel segments were functionally denuded of endothelium [5]. The preparations were exposed to cumulatively concentrations of tested compounds (10^−12^–10^−3^ M) added to the preparations until a maximum response was observed as indicated by a plateau response (approximately 3–5 min).

### 4.4. Statistical Analysis

The results were expressed as mean ± standard error of the mean (SEM). Differences between means were considered significant when *p* < 0.05. Statistical comparisons between two variables were performed by using Student’s *t*-test unpaired. The results were analyzed and plotted using GraphPad Prism 5.0 software (GraphPad Software**,** San Diego, CA, USA).

## 5. Conclusions

The study of *S. rhombifolia* aerial parts, using chromatographic and spectroscopic techniques, led to the identification of eight substances, among them two coumarins: scopoletin (**1**) and escoporone (**2**); a ferulic acid derivative, ethoxy-ferulate (**3**); two flavonoids: kaempferol (**4**) and kaempferol-3-*O*-β-d-glycosyl-6′′-α-d-rhamnose (**5**); and three indoquinoline alkaloids: quindolinone (**6**), 11-methoxy-quindoline (**7**) and quindoline (**8**). Compound **3** is being first reported in the *Sida* genus, and others have been previously reported strengthening *Sida* chemosystematics [23,24,25].

The quindolinone (**6**) and the salt of cryptolepine (**9**) induced vasorelaxation dependent on the vascular endothelium, justifying the use of the species in popular medicine in India. The vasorelaxation effect may be related to indoquinoline core, but more protocols are required to elucidate their complete mechanism of action.

## Figures and Tables

**Table 1:** NMR data (^1^H, ^13^C, HMQC, HMBC and NOESY) of compound ***7*** (δ, DMSO-*d*_6_, 500 and 125 MHz).

H/C	HSQC		HMBC			NOESY
	δ_H_	δ_C_	^2^*J*	^3^*J*	^4^*J*	
1	8.30 (bd, *J* = 8.45 Hz, 1H)	121.3	-	C-11/C-4a/C-3	-	OCH_3_/H-2
2	7.58 (bt, *J* = 7.55 Hz, 1H)	124.5	-	C-4/C-11a	-	H-1
3	7.70 (bt, *J* = 7.45 Hz, 1H)	127.1	-	C-4a	-	H-4
4	8.18 (bd, *J* = 8.5 Hz, 1H)	127.9	-	C-2/C-11a	-	H-3
4a	-	143.9	-	-	-	-
5-N	-	-	-	-	-	-
5a	-	126.7 *	-	-	-	-
6a	-	121.7	-	-	-	-
6	8.37 (bd, *J* = 7.85 Hz, 1H)	121.4		C-9a/C-8		H-7
7	7.30 (bt, *J* = 7.02 Hz, 1H)	119.6	C-8/C-6	C-9	C-9a	H-6/H-8
8	7.63 (bt, *J* = 7.6 Hz, 1H)	130.0	C-7	C-9a	-	H-7
9	7.64 (bd, *J* = 7.1 Hz, 1H)	112.0	C-9a	C-7	-	-
9a	-	143.9	-	-	-	-
10-NH	11.50 (s, 1H)	-	-	-	-	OCH_3_/H-9
10a	-	125.6 *	-	-	-	-
11	-	146.1	-	-	-	-
11a	-	120.3	-	-	-	-
OCH_3_	4.39 (s, 3H)	60.7	-	C-11	-	H-1/10-NH

* The values may be interchanged: 5a-125.6; 10a-126.7.
